# Enhancing anandamide signalling through fatty acid amide hydrolase inhibition: An update on the pharmacological strategy for treating psychiatric disorders

**DOI:** 10.1038/s41398-026-04120-4

**Published:** 2026-05-28

**Authors:** Timothy A. Couttas, Anna E. Hoffmann, Beverly Jieu, Felix R. Golla, Claire E. Shepherd, F. Markus Leweke, Cathrin Rohleder

**Affiliations:** 1 Department of Psychiatry and Psychotherapy, Central Institute of Mental Health, Medical Faculty Mannheim, Heidelberg University, Mannheim, Germany; 2 Neuroscience Research Australia, Randwick, NSW Australia; 3 Brain and Mind Centre, Faculty of Medicine and Health, The University of Sydney, Sydney, NSW Australia; 4 Endosane® Pharmaceuticals GmbH, Berlin, Germany

**Keywords:** Psychiatric disorders, Drug discovery

## Abstract

Endocannabinoids (eCBs) are lipid-derived neuromodulators that regulate numerous neurophysiological processes by modulating synaptic transmission. Synthesised on demand in response to increased postsynaptic intracellular calcium or activation of postsynaptic G-protein coupled receptors, eCBs are rapidly degraded, resulting in transient, tightly regulated signalling. Dysregulation in the endocannabinoid system (ECS), including altered peripheral and central eCB concentrations and/or cannabinoid-1 receptor (CB_1_R) expression, has been observed across psychiatric syndromes, including major depressive disorder, psychotic disorders, and post-traumatic stress disorder (PTSD). These associations have prompted growing interest in pharmacological strategies targeting the ECS. Though medical cannabis is increasingly prescribed for psychiatric symptoms, its clinical use remains controversial due to limited high-quality evidence, psychotropic side effects, and regulatory constraints. An alternative is to enhance the signalling of a principal eCB, anandamide (AEA), potentially offering more physiologically constrained CB_1_R engagement, by inhibiting fatty acid amide hydrolase (FAAH), the main enzyme degrading AEA and its congener, N-acylethanolamines (NAE), oleoylethanolamide (OEA) and palmitoylethanolamide (PEA). This review consolidates recent clinical evidence for FAAH inhibitors, examining their influence on AEA, safety and efficacy in ameliorating symptoms across a range of psychiatric conditions, including depression, anxiety, PTSD, and cannabis use disorder (CUD). Presently, only two compounds, PF-04457845 (JZP150) and JNJ-42165279, have progressed to Phase II trials, demonstrating modest clinical benefit in CUD, with no efficacy in PTSD or osteoarthritis pain. Herein, we discuss emerging insights, safety considerations, broader mechanistic implications, and future directions for FAAH-targeted therapeutics, advocating for a precision medicine approach to realise their potential in the treatment of psychiatric disorders.

## Introduction

**Fig. 1:**
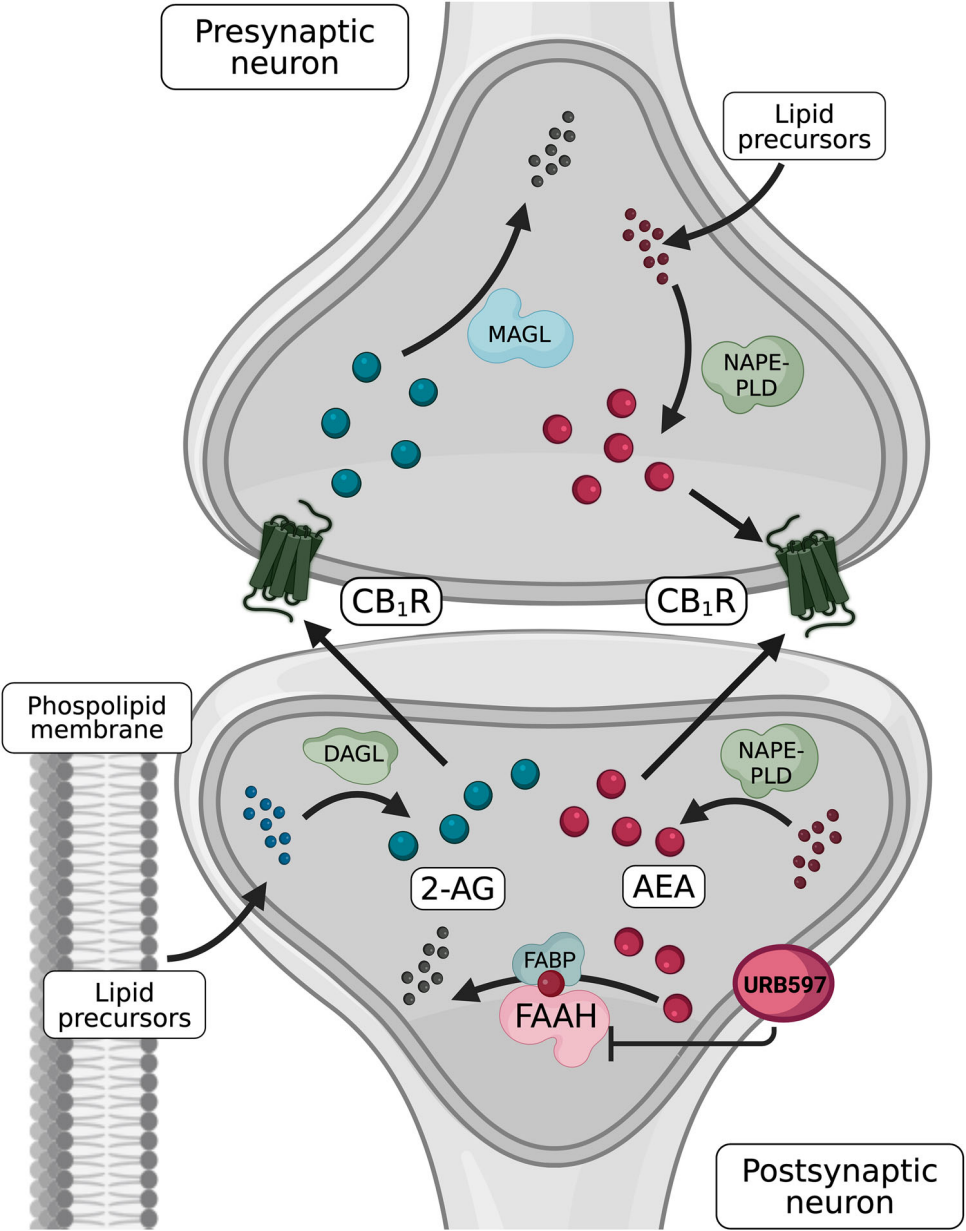
Schematic overview of eCB signalling and FAAH mediated modulation. AEA and 2-AG are synthesised on demand from membrane phospholipid precursors, primarily via the N-arachidonoyl phosphatidylethanolamine specific phospholipase D (NAPE-PLD) pathway for AEA, which appears to occur in both pre and postsynaptic neurons, and via diacylglycerol lipase (DAGL) for 2-AG. Following synthesis, AEA is transported intracellularly by fatty acid binding proteins (FABPs), noting other AEA transporters have been suggested (e.g., heat shock protein 70, albumin, sterol carrier protein 2), which facilitate its retrograde trafficking. AEA is subsequently hydrolysed by FAAH into arachidonic acid and ethanolamine, while 2-AG is broken down by monoacylglycerol lipase (MAGL). Pharmacological inhibition of AEA hydrolysis is depicted via URB597, a selective FAAH inhibitor that elevates AEA. These mechanisms modulate synaptic eCB tone and represent key targets for therapeutic intervention. Note: Though this schematic focuses on the canonical NAPE-PLD and FAAH pathways, AEA signalling also involves additional biosynthetic (e.g., α,β-hydrolase domain-containing 4, ABHD4; glycerophosphodiesterases 1, 4, or 7, GDE1, GDE4, GDE7) and degradative (e.g., N-acylethanolamine acid amidase (NAAA); cyclooxygenase-2 (COX-2); lipoxygenases) pathways. These have not been depicted to maintain visual clarity. Created in BioRender. Rohleder, C. (2026) https://BioRender.com/ppn0frn.

The endocannabinoid system (ECS) is widely distributed throughout the peripheral and central nervous system (CNS), various organs, and the immune system, where it is recognised for maintaining neurological and physiological homeostasis [1]. Within the CNS, the ECS is implicated in the pathogenesis of mood, anxiety, trauma-related, and psychotic conditions, including major depressive disorder (MDD), schizophrenia, and post-traumatic stress disorder (PTSD) [2–5]. The ECS comprises two principal G protein-coupled cannabinoid receptors (CB_1/2_R). CB_1_R is predominantly expressed in the CNS (notably in the cortex, basal ganglia, hippocampus and cerebellum), though also present in peripheral tissues (e.g., liver, adipose tissue and skin), while CB_2_R is primarily expressed on immune cells, including microglia, B cells, but also in natural killer (NK) cells, monocytes, neutrophils, CD8^+^ T cells, and at low levels on CD4^+^ T cells, consistent with a role in immune activation and inflammatory signalling [1, 6–9]. As reviewed by Lu and Mackie, within the CNS, CB_1_R is most abundant on specific GABAergic (γ-aminobutyric acid) interneurons but is also expressed across other, diverse, neuronal populations, including glutamatergic, cholinergic, glycinergic, and serotonergic neurons [1]. In neurons, CB_1_R is particularly enriched at presynaptic terminals, consistent with its key role in regulating synaptic transmission; however, expression is also observed at significant levels on neuronal somata, dendrites, and some mitochondria [1]. These receptors are activated by endocannabinoids (eCBs), endogenous lipid-derived ligands produced on demand from membrane lipid precursors in response to cellular signals such as postsynaptic G-protein activation or calcium influx [1, 10]. The most studied eCBs are *N-*arachidonoylethanolamide (anandamide, AEA; a partial agonist at CB_1_R with very low efficacy at CB_2_R) and 2-arachidonoyl-*sn*-glycerol (2-AG; a full agonist at CB_1_R and CB_2_R) [11–13], both arachidonic acid derivatives, forming part of a larger lipid-derived network implicated in major mood, anxiety- and trauma-related, and psychotic disorders [14, 15]. Although both share the same fatty acid precursor, AEA and 2-AG biosynthesis and degradation are regulated by distinct enzymatic pathways [1, 13]. An overview of the synthesis, transport and degradation of both neuronal signalling eCBs is depicted in Fig. 1. Functionally, 2-AG is often considered the principal mediator of rapid, activity-dependent retrograde synaptic signalling, whereas AEA may preferentially contribute to modulatory “eCB tone”, defined as the baseline level of eCB signalling within a given tissue or circuit, that is governed by the balance between synthesis/release and uptake/degradation [16]. Therefore, alterations to AEA’s steady state may have a greater influence on longer-term regulation within neural networks [17].

### Alterations to AEA in mood, anxiety, trauma-related, and psychotic conditions

AEA has drawn particular interest in psychiatric research due to its strong affinity for CB_1_R, which is itself implicated in various neuropsychiatric disorders [18–20]. Initially, elevated concentrations of AEA have been observed in the cerebrospinal fluid (CSF) of individuals diagnosed with schizophrenia, where AEA appears to display protective properties early in the pathogenesis [21–25]. *Cannabis sativa* (cannabis) use is a recognised risk factor for the development and relapse of psychosis [26–28], and may attenuate this compensatory rise in AEA. For instance, among individuals with schizophrenia, CSF AEA concentrations were ~10-fold higher in low-frequency, compared to high-frequency cannabis users, suggesting that sustained cannabis exposure may blunt the compensatory CSF AEA elevation reported in schizophrenia [29]. In addition, individuals presenting with psychotic symptoms who tested positive for delta-9-tetrahydrocannabinol (Δ^9^-THC) displayed lower AEA in blood plasma than those testing negative [30]. Similarly, heavy cannabis use in otherwise healthy individuals is associated with lower CSF AEA [31], while individuals with cannabis use disorder (CUD) cessation attempts have been linked to reduced plasma AEA, a decrease which was prevented by the phytocannabinoid cannabidiol (CBD), the primary non-psychotomimetic constituent of cannabis [32].

Garani et al., provided a comprehensive review of eCB disruption in mood-related disorders [3], highlighting a significant reduction of AEA, in the sera of individuals who experienced a major depressive episode or MDD, with these eCBs correlating to symptom severity [33, 34]. Reduced concentrations of the AEA metabolite, ethanolamine, have also been reported in the CSF of individuals with MDD, with higher ethanolamine observed in individuals with remitted compared to non-remitted MDD and healthy controls [35]. Nonetheless, conflicting reports indicate that AEA is associated with the anxiety dimension of mood disorders [34], or with moderate depression rather than mild depression, but not with the severity of depression per se [36]. Moreover, elevated AEA alongside its congener oleoylethanolamide (OEA) correlated with increases in somatic symptoms, including changes in appetite, circadian rhythm, and anhedonia [36]. Acute psychosocial stress (Trier Social Stress Test) has been shown to increase serum AEA immediately following stress exposure in healthy controls; however, a sub-analysis revealed AEA increase reached statistical significance only in men [37]. This finding aligns with a separate study that exposed women with MDD and matched female controls to the same stress paradigm and found no changes in serum AEA in response to stress [33]. Elevated AEA tone has similarly been associated with reduced fear and stress sensitivity in humans. Individuals carrying the C385A variant of fatty acid amide hydrolase (FAAH), the principal enzyme involved in AEA hydrolysis, show reduced FAAH activity and increased AEA, resulting in enhanced fronto-amygdala connectivity, improved fear extinction learning, and lower trait anxiety levels [38]. Similarly, high baseline serum AEA concentrations were associated with low anxiety ratings in healthy controls [37].

The ECS is also thought to modulate stress responses by inhibiting the hypothalamic-pituitary-adrenal (HPA) axis via CB_1_R signalling in corticolimbic circuits [39, 40]. Consequently, it has been implicated in PTSD, whose pathophysiology is often characterised by dysregulation of the HPA axis [41]. In further support of a modulatory role for the ECS in PTSD, individuals carrying the FAAH C385A allele with comorbid PTSD and alcohol dependence have shown reduced subjective anxiety in response to a stress challenge [42]. These individuals also reported lower levels of PTSD-related hyperarousal over the course of the study compared to C allele homozygotes. However, studies reporting AEA in PTSD remain inconsistent, noting unaltered [43–45], alongside elevated and reduced AEA blood concentrations compared to controls [46–48], likely reflecting clinical heterogeneity (e.g., symptom profiles, comorbid symptoms, and trauma characteristics, including type and duration), as well as sex-related and methodological factors (e.g., sample size, and assay differences). In their pooled analysis, Botsford and colleagues reported no significant differences in circulating (i.e., blood) AEA concentrations among women with or without a PTSD-related diagnosis [49]. However, they noted that lower baseline AEA within their PTSD cohorts was associated with greater depressive symptom severity, confusion, total mood disturbance, and the PTSD “negative alterations in cognition and mood” cluster, suggesting that despite heterogeneous findings across studies, increasing AEA may support improvement toward specific symptom dimensions in PTSD [49].

### Therapeutic approaches targeting AEA

Despite advances in pharmacotherapy, treatment options remain limited across many psychiatric conditions, e.g., PTSD, borderline personality disorder (BPD), anxiety disorders, and schizophrenia, although non-pharmacological approaches are helpful in the first three conditions, underscoring the need for novel therapeutic strategies. For PTSD, only two specific drugs, sertraline and paroxetine, have been approved in the US and Europe, and they are non-specific. Pharmacotherapy for BPD is symptom-targeted and based only on off-label use of other compounds. For anxiety disorders, effective medications are mainly derived from antidepressant classes, while agents like pregabalin have been proven effective, along with tranquilizers, which are effective but have significant abuse and addiction risk. In schizophrenia, most approved compounds act via similar mechanisms, cause substantial side effects, and are only partially effective or even ineffective in 19.8% of cases.

One potential novel pharmacotherapeutic strategy is to target the ECS. Cannabis, including medical preparations and pure cannabinoids, has been widely touted in observational studies for its ability to alleviate symptoms of psychosis, mood and anxiety disorders, and PTSD [50]. Medical cannabis is frequently prescribed for anxiety disorders [51], a practice largely driven by observational findings and reports claiming reductions in anxiety and depressive symptoms [52]. Randomised clinical trials (RCTs) investigating these indications remain scarce [53, 54], presumably owing to concerns regarding cannabis abuse liability and potential symptom exacerbation [55, 56]. Given that the cannabis effects are mediated through the ECS, efforts have shifted toward the development of pharmacological agents that target this system, while offering improved efficacy and safety.

Among the cannabis-derived compounds, CBD is considered a promising phytocannabinoid therapeutic. Clinical evidence suggests it exerts clinical benefits both as a monotherapy and adjunctive treatment in schizophrenia [57, 58]. These effects are accompanied by dose-dependent increases in AEA signalling [57, 59]. Though Δ^9^-THC is a partial agonist at CB_1/2_R, CBD exhibits low affinity for CB_1/2_R, with evidence indicating that CBD elevates AEA through moderate inhibition (μM range) of FAAH [60–62]. This mechanism has been verified in several preclinical models [57, 63, 64]. In humans, however, CBD does not appear to directly inhibit FAAH; rather, it enhances AEA concentrations indirectly by preventing the intracellular transport of AEA to FAAH via fatty acid binding proteins (FABPs) [65]. An additional hypothesis proposes that CBD may stimulate AEA synthesis by activating the enzyme NAPE-PLD, though this mechanism remains insufficiently characterised [66].

Given accumulating evidence that AEA dysregulation is involved in psychiatric conditions, alternative approaches have focused on directly enhancing this eCB's tone rather than targeting CB_1_Rs themselves. This can be achieved by targeting transport proteins or the enzymes responsible for eCB synthesis or degradation. This review focuses on the inhibition of FAAH as a means to prevent AEA degradation and thus enhance ECS signalling. Although both humans and primates express two isoforms of FAAH, sharing approximately 20% sequence homology [62], pharmacological inhibitors have primarily targeted the predominant isoform, commonly referred to as FAAH, due to its higher affinity for AEA hydrolysis [62, 67].

FAAH inhibitors have been designed from various chemical classes, including trifluoromethylketones, α-keto esters and amides, bromoenol lactones, non-steroidal anti-inflammatories and fluorophosphonates [68, 69]. However, as Piomelli et al. noted, these inhibitors often lack the selectivity and/or bioavailability essential for therapeutic application in vivo [70]. Second-generation carbamates, derived from an N-cyclohexylcarbamic acid O-aryl ester template, covalently modify the active site to inhibit FAAH activity with nanomolar potency and nil affinity for CB_1_R or other cannabinoid targets [71–73]. Preclinical testing has shown FAAH inhibitors improve outcomes in models of schizophrenia and PTSD [74], exhibit analgesic and anxiolytic effects [71, 75], and may benefit individuals with allodynia, epilepsy and depression [76–78]. These promising preclinical findings have prompted several clinical trials using FAAH-selective inhibitors, as reviewed by van Egmond et al. [79] and Mayo et al. [80]. This review updates recent pharmacological advancements, including FAAH-inhibitor progression in RCTs and emerging evidence on their safety, tolerability, efficacy and therapeutic potential toward psychiatric and mood-related disorders.

## FAAH inhibitors in clinical practice

**Fig. 2:**
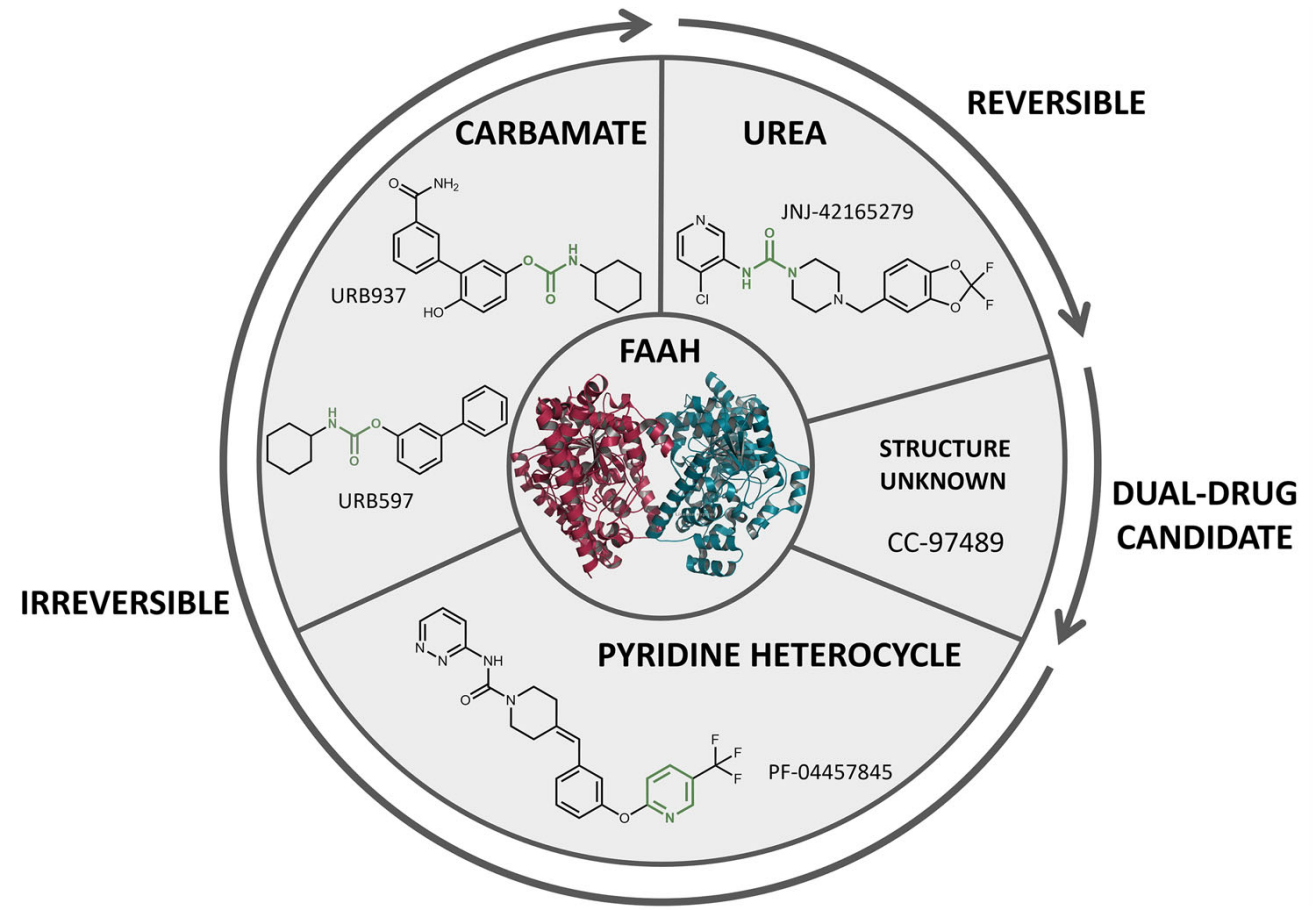
FAAH inhibitors currently undergoing RCTs, categorised (where available) by the structural motifs (highlighted in green) used in their design and type of inhibition (reversible, irreversible or dual FAAH/MAGL inhibitor). Created in BioRender. Rohleder, C. (2026) https://BioRender.com/d0n7ofq.

To summarise recent clinical developments in FAAH-selective inhibitors, we performed a targeted review of published literature and clinical trial registries. PubMed and Scopus were searched using terms including *FAAH inhibitor*, *clinical trial*, *anandamide/AEA*, and *endocannabinoid/eCB*, as well as names of individual compounds (e.g., URB597, PF-04457845, JNJ-42165279) with filters for English-language, human studies from January 2022 to August 2025. Additional information on trial design, dosage, safety, and outcomes was sourced from ClinicalTrials.gov, the European Union Drug Regulating Authorities Clinical Trials Database (EudraCT), and the EU Clinical Trials Register (Table 1), with structures of the individual compounds represented, where available (Fig. 2). We prioritised peer-reviewed sources where possible, noting that grey literature from press releases, corporate communications and trial registries was also incorporated where peer-reviewed data was unavailable or for ongoing trials, which has been explicitly stated in text. For greater details on FAAH-selective inhibitors and clinical investigations either terminated or discontinued before 2022, we refer readers to prior reviews [79, 81, 82].

**Table 1:** Updates to FAAH inhibitors currently undergoing or recently completed randomised clinical trials relevant to psychiatric and mood disorders.

Clinical Trial	Treatment/Study aim	Study Design	Status	Efficacy & Safety Outcomes	Ref
***PF-04457845 (JZP150)***					
NCT03386487	Cannabis use disorder	Phase IIb, double-blind, placebo-controlled, parallel-group; 8-week RCT. Study arms: PF-04457845: (4 mg/day, n = 112), placebo (n = 116).	Completed (2022)	*Efficacy* Timeline follow back for self-reported cannabis use: • Baseline: 1.921 (drug); 1.768 (placebo) • Week 8: 1.113 (drug); 0.936 (placebo) Urinary THC-COOH (ng/ml): • Baseline: 5.959 (Drug); 5.571 (placebo) • Week 9: 3.222 (Drug); 3.952 (placebo) *Safety* Adverse responses similar between placebo and drug group, noting dizziness and depressed mood marginally higher with drug-treatment. Two serious adverse events (SAEs, 1.72%) reported for drug-treatment: bowel obstruction and abscess removal. Total of AEs in 76.72% and 81.25% of participants of the PF-04457845 and placebo group, respectively.	N/A
NCT05178316	Post-traumatic stress disorder	Phase II, double-blind, placebo-controlled, parallel-group; 12-week RCT. Study arms: JZP150 (0.3 or 4 mg/day), placebo, n = 282 enrolled.	Completed (2023)	*Efficacy* No statistically significant decrease in symptom severity, as assessed by Clinician Administered PTSD Scale (CAPS-5), Clinical Global Impression of Severity (CGI-S) and Patient Global Impression of Severity (PGI-S). *Safety* 1 SAE (1.85%, seizure) in the 0.3 mg/day group, 2 SAEs (1.77%, blood potassium decreased, dehydration) in the 4 mg/day group, 1 SAE (0.91%, pneumonia staphylococcal infections with sepsis) in the placebo group. Total of AEs in 37.04%, 26.55%, and 21.82% of the participants of the 0.3 mg/day, 4 mg/day and placebo group, respectively.	[96]
***JNJ-42165279***					
NCT03664232	Autism spectrum disorder (ASD)	Phase II, double-blind, placebo-controlled, parallel group; 12-week RCT. Study arms: JNJ-42165279 (2 × 25 mg/day), placebo, n = 78 enrolled.	Completed (2022)	*Efficacy* Change from Baseline in Autism Behaviour Inventory (ABI) at day 85: • ABI-Core Domain: −0.26 ± 0.274 (drug) −0.20 ± 0.400 (placebo), p = 0.284 • ABI Social Communication, 0.27 ± 0.332 (drug) −0.22 ± 0.379 (placebo), p = 0.290 • ABI Repetitive/Restrictive Behaviour 0.23 ± 0.299 (drug) −0.17 ± 0.525 (placebo) (p = 0.231) Although JNJ-42165279 did not significantly reduce ASD symptoms assessed as part of the primary outcome, moderate changes favouring the drug were observed for a few secondary outcomes (Repetitive Behaviour Scale Revised (p = 0.006), Child Adolescent Symptom Inventory-Anxiety (p = 0.048) *Safety* No serious adverse events were reported. Total of AEs in 46.9% and 40.0% of the participants of the JNJ-42165279 and placebo group, respectively.	[102]
EudraCT 2020-001965-36	Post-traumatic stress disorder	Phase I/II, double-blind, placebo-controlled, parallel group; 12-week RCT with internet-delivered cognitive behavioural therapy (iCBT). Study arms: JNJ-42165279 (2 × 25 mg/day, n = 45), placebo, n = 41 All participants completed iCBT (week 5–12). 100 enrolled (85 women, 15 men), with 14 dropouts.	Completed (2021)	*Efficacy* Significant main effect of reduced PTSD symptom severity (structured interview CAPS-5, primary outcome) over course of study. Improvement in self-reported symptoms (secondary outcomes assessed using posttraumatic stress disorder checklist for DSM-5 (PCL-5), Comprehensive Psychopathological Rating Scale, Self-Administered (CPRS-S-A, anxiety and depression subscale). Sleep quality (assessed using Pittsburgh Sleep Quality Index (PSQI)), remained stable during the RCT. Symptom improvements (CAPS-5) were not specific to JNJ-42165279 administration. No direct effect from JNJ-42165279 toward any secondary measures (PCL-5, CPRS-S-A, PSQI). Plasma concentrations of JNJ-42165279 significantly correlated with AEA (r_s_ = 0.67, p < 0.001). Baseline AEA showed no main effect on symptom severity. Study concluded no enhanced response from JNJ-42165279 when used as an adjunct treatment with iCBT. *Safety* Two SAEs in the placebo group. Total of AEs in 80% and 82.0% of the participants of the JNJ-42165279 and placebo group, respectively.	[101]
NCT02498392	Major depressive disorder, anxiety	Phase IIa, double-blind, placebo-controlled, parallel-group; 18-week RCT. Study arms: JNJ-42165279 (25 mg/day for 6 weeks, n = 77), placebo (n = 76)	Completed (2019)	*Efficacy* Change from Baseline in Hamilton Depression Rating Scale (HDRS17) at Week 6: • eITT: −6.5 ± 4.0 (drug); −6.1 ± 5.9 (placebo) • fITT: −5.2 ± 4.7 (drug); −5.0 ± 6.3 (placebo) *Safety* Two severe adverse events were reported in the placebo group (1.25%; gastroenteritis and foot deformity) and another with drug-treatment (1.30%; foot deformity). Total of AEs in 3.90% and 5.26% of the participants of the JNJ-42165279 and placebo group, respectively. All AEs were headaches.	N/A
***CC-97489 (BMS-986368)***					
NCT06227975	Pharmacokinetics	Phase I, open-label; 30-day single group assignment. Study arms: [14 C]-BMS-986368 (Dose not specified, n = 8)	Completed (2024)	Results yet to be published.	N/A
NCT05065541	Evaluate enzyme availability in the central nervous system.	Phase I, open-label, Positron Emission Tomography; 3-week non-randomised. Study arms: [11 C]MK-3168 and [18 F]T-401 PET ligands received before and after oral administration CC-97489 (single and multiple dosages, unspecified, n = 32)	Completed (2024)	Results yet to be published.	N/A
NCT06411730	Safety, tolerability, pharmacokinetics, pharmacodynamics	Phase I, double-blind, placebo-controlled; 3-week RCT. Study arms: BMS-986368 at ascending doses (not specified), placebo; n = 64 estimated to be enrolled.	Completed (2024)	Results yet to be published.	N/A
NCT05099822	Safety, tolerability, pharmacokinetics, pharmacodynamics	Phase I, double-blind, placebo-controlled, single and multiple-ascending doses; 4-week RCT. Study arms: CC-97489 at single and multiple ascending doses (not specified), placebo; n = 84 enrolled but not assigned.	Terminated (2022)	Terminated due to change in business objectives	N/A
***URB597 (KDS-4103)***					
NCT00916201	Schizophrenia	Phase I, double-blind, parallel group; 1-week RCT. Study arms: URB597 (10 mg/day, for 5 days), Cannabidiol (320 mg/day, for 5 days), Insulin intranasal (160 IU/day, for 5 days); n = 86 estimated to be enrolled but not yet assigned.	Withdrawn (2025)	Withdrawn due to lack of funding.	N/A

*fITT*, full intent to treat; *eITT*, enriched intent to treat; *N/A*, no literature reference available.

### URB597

URB597 (KDS-4103), an alkylcarbamic acid aryl ester developed in partnership between the Universities of California-Irvine, Urbino and Parma [72], is among the most extensively investigated FAAH-selective inhibitors. In vitro pharmacology assessments demonstrated irreversible selective inhibition of FAAH, without affecting neurotransmitter transporters, serine hydrolases, CB_1/2_R, cytochrome P450 isoforms, HERG channel activity or ion channels, as reviewed in [70]. Preclinical investigations unveiled a significant, dose-dependent inhibition of FAAH activity in brain homogenates, reaching half-maximal inhibition at approximately 0.15 mg/kg in rats [71]. Intraperitoneal administration (i.p.) at 0.3 mg/kg produced rapid (<15 min) and sustained (>12 h) FAAH inhibition in both cerebral and peripheral (liver, duodenum) tissue homogenates, accompanied by marked increases of AEA and congener ethanolamine’s OEA and palmitoylethanolamine (PEA) [83].

Preclinical safety studies supporting the Investigational New Drug (IND) application commenced in 2006, as reviewed in [70]. URB597 displayed no systemic toxicity from single oral doses up to 2000 mg/kg in rats, over 40 times the required dose for significant inhibition, and up to 1500 mg/kg in primates. Repeated dosing (1500 mg/kg) in rats (28 days) or non-human primates (21 days) showed no treatment-related clinical observations and no evidence of toxicity in blood chemistry, haematology or gross necropsy. CNS safety assessments, involving a comprehensive functional observation battery in rats, demonstrated no apparent effects at oral doses up to 1500 mg/kg. Furthermore, in rats, the rotarod test indicated no motor impairments at doses (5 mg/kg i.p.), substantially (33-fold) exceeding the median effective dose (ED_50_) for brain FAAH inhibition. Bacterial cytotoxicity (in vitro) and Ames testing were also negative.

Self-administration studies indicated that URB597 has no abuse liability in rodents [73, 84, 85], with analogous results observed in squirrel monkeys, which did not self-administer URB597 across a range of doses [86]. Furthermore, URB597 did not reinstate extinguished drug-seeking behaviour in paradigms previously maintained by Δ^9^-THC, AEA, or cocaine [86]. In contrast, exogenous AEA can sustain intravenous self-administration behaviour in squirrel monkeys in a CB_1_R-dependent manner, supporting a role for AEA signaling in brain reward-related processes under conditions of rapid, pharmacological CB_1_R activation [87]. However, intravenous or intraperitoneal injections of AEA did not elicit typical abuse-related behaviours in conditioned place preference or aversion paradigms, which are commonly used to study drug reward in animals [85, 88, 89]. Mechanistically, two non-mutually exclusive explanations have been proposed to account for the lack of reinforcing effects of URB597 in primates despite enhanced AEA signalling [86]. Firstly, endogenous AEA elevation may preferentially access a CB_1_R pool distinct from that recruited by exogenous AEA, which may be accompanied by compensatory reductions in 2-AG signalling, thereby attenuating reinforcement. Secondly, FAAH inhibition may result in slower CB_1_R recruitment than direct AEA administration, whereas effective reinforcement depends strongly on rapid drug distribution and receptor engagement. Collectively, the current data indicates URB597 may offer therapeutic benefit without relevant abuse or relapse potential.

To date, only one clinical trial involving URB597 has been registered on ClinicalTrial.gov (NCT00916201). This interventional clinical evaluation (Phase I) aimed to compare the effects of URB597, CBD and intranasal insulin to treat/ameliorate symptoms of schizophrenia. Recently, this trial was withdrawn due to insufficient funding to support transition to the new Clinical Trial Regulations.

### PF-04457845

Compound 23, originally named PF-04457845 in Johnson et al. [90], is a highly selective, irreversible FAAH inhibitor that carbamylates the serine nucleophile active-site, with nanomolar potency (IC_50_ of 7.2 nM for human FAAH in vitro). Oral administration demonstrated antinociceptive effects in acute inflammatory and non-inflammatory pain rodent models (based on complete Freund’s adjuvant solution and monosodium iodoacetate, respectively), as well as reduced FAAH activity and elevated concentrations of AEA in both plasma (3 to 5-fold) and brain membrane fractions (5 to 7-fold) from humans and mice [91]. In Phase I RCTs, PF-04457845 displayed excellent tolerability at doses exceeding the concentrations required for maximal FAAH inhibition [92]. In 2012, Pfizer halted the development of PF-04457845 after a Phase II trial failed to relieve knee osteoarthritic pain despite modulating AEA and related NAEs [93]. However, a concurrent trial initiated prior to discontinuation reported that PF-04457845 reduced cannabis use and withdrawal symptoms in men, as evidenced by self-report, lower urinary concentrations of Δ^9^-THC and its carboxylic acid metabolite (Δ^9^-THC-COOH), as well as decreased scores on the Marijuana Withdrawal Checklist [94].

Pfizer also terminated an evaluation of PF-04457845’s effects on BOLD fMRI signals in subjects with PTSD (NCT02216097), due to portfolio reprioritisation rather than safety or efficacy concerns. In 2014, a Phase I RCT was initiated to examine the safety, tolerability, and feasibility of PF-04457845 (4 mg per day over 4 weeks) in adults with Tourette syndrome (NCT02134080). According to ClinicalTrials.gov, this study was terminated due to insufficient funding. Additionally, Pfizer provided PF-04457845 for an investigator-initiated, double-blind, placebo-controlled trial (EudraCT Number: 2016-005013-47), assessing its effects (4 mg, orally once daily for 10 days, n = 16; placebo, n = 29) on fear learning, stress reactivity, and stress-induced responses in healthy volunteers. PF-04457845 induced a 10-fold increase in baseline AEA plasma that was associated with potentiated recall of fear extinction memory, attenuated autonomic stress reactivity, and protected against stress-induced negative effects, without impacting baseline mood or anxiety [95]. The authors concluded that PF-04457845 may have therapeutic potential for PTSD.

At present, a Phase IIb RCT is underway evaluating the efficacy, safety and tolerability of PF-04457845 in adults with current Cannabis Use Disorder (NCT03386487). Led by D’Souza et al., who previously reported PF-04457845 clinical benefits in men with cannabis dependence [94], the RCT’s primary outcomes were updated in February 2024 (Table 1), though no statistical results have been published to date. Available summaries indicate that self-reported cannabis use decreased to a similar extent in both the PF-04457845 and placebo groups, while urinary THC-COOH concentrations decreased in both groups, with a numerically larger reduction in the PF-04457845 group. A separate Phase II trial investigating PF-04457845 effects on fear conditioning (NCT01665573) was completed in 2022, but no updates on this RCT are available since earlier reviews [95].

Jazz Pharmaceuticals acquired PF-04457845, now called JZP150, from SpringWorks Therapeutics, who licensed the product from Pfizer in 2020. In a Phase II RCT, 282 adult participants (18 to 70 years) diagnosed with PTSD (NCT05178316) received either 0.3 mg or 4 mg doses of JZP150 or placebo, over a 12-week period. However, according to a press release, JZP150 failed to show a statistically significant decrease in the severity of symptoms over placebo, missing the trial’s primary (Clinician Administered PTSD Scale, CAPS-5) and secondary (Global Impression of Severity and the Patient Global Impression of Severity) endpoints, and was discontinued [96].

### JNJ-42165279

JNJ-42165279 is an aryl piperazinyl urea compound, developed by Janssen Pharmaceuticals, which inhibits FAAH (IC_50_ human recombinant FAAH = 70 nM; rodent FAAH = 313 nM) by covalent bonding to the catalytic site [97]. The inhibition is reversible, via slow hydrolysis of the bound drug fragment. Preclinical studies showed JNJ-42165279 to be highly potent and selective in blocking FAAH activity, resulting in >4-fold increase of baseline AEA in the brain, with associated benefits in rodent models of neuropathic pain and inflammation [97]. In Single (NCT01650597) and Multiple Ascending Dose (NCT01964651, NCT02169973) Phase I safety studies, the compound was well-tolerated, exhibiting no severe adverse effects, and demonstrated robust central and peripheral FAAH inhibition at doses ≥10 mg [98]. Single doses of 10–100 mg produced mean peak plasma AEA concentrations that were ~5.5–10-fold higher compared to placebo. Daily dosing increased CSF AEA by ~45-fold (10 mg), ~41-fold (25 mg), and ~77-fold (75 mg) relative to pre-dose values [98]. These pharmacodynamic effects were accompanied by 96–98% of brain FAAH occupancy, as assessed by a positron emission tomography (PET) scanning (radiotracer: [^11^C]MK3168) after single doses of 10–50 mg, indicating near-complete brain enzyme saturation [98].

Janssen Pharmaceuticals voluntarily halted development of JNJ-42165279, as a precaution following the serious incidents with BIA 10-247 (see below). After a safety review indicating only a very minor risk for serious adverse events [82], trials resumed in 2018. In a Phase IIa RCT, JNJ-42165279 (25 mg daily) demonstrated moderate anxiolytic effects in social anxiety disorder [99], with over 30% improvement on the Liebowitz Social Anxiety Scale (LSAS), and superior Clinical Global Impression-Improvement (CGI-I) scores by the end of treatment (12 weeks) [99]. However, JNJ-42165279 did not significantly outperform placebo on the LSAS primary endpoint or the Sheehan Disability Scale [99]. A parallel RCT showed that a 4-day treatment with 100 mg (n = 43) dampens responses to emotional stimuli, but not to conditioned fear [100]. In a 12-week double-blind RCT in individuals with PTSD (EudraCT 2020-001965-36), adjunctive treatment with JNJ-42165279 (25 mg b.i.d) did not enhance the efficacy of internet-delivered cognitive behavioural therapy (iCBT, eight weekly modules starting in week 5 of medication treatment) compared to iCBT plus placebo. No significant differences were observed between groups in the structured interview “clinician-assessed PTSD symptom severity (CAPS-5)”, self-reported PTSD symptoms, anxiety, depression, or sleep quality, despite a significant increase in plasma AEA [101].

Recent updates to a Phase II RCT for JNJ-42165279 treatment of MDD and anxiety (NCT02498392, Table 1) showed no benefit towards the primary outcome – the change from baseline in the Hamilton Depression Rating Scale (HDRS17) scores – or the secondary measures including baseline changes in the Hamilton Anxiety Rating Subscale (HAM-A6) and the Structured Interview Guide of the Hamilton Anxiety Scale (SIGH-A). A separate Phase II RCT investigating the efficacy, safety, and tolerability of JNJ-42165279 in adolescents and adults with autism spectrum disorder was completed in October of 2022 (NCT03664232). Although JNJ-42165279 did not demonstrate superiority over placebo towards the primary outcomes, including change in the Autism Behaviour Inventory (ABI) Core Domain Score, ABI-Social Communication, and ABI-Restrictive Behaviour, significant improvements were observed in several secondary outcomes, including a greater reduction in repetitive behaviour (Repetitive Behaviour Scale – Revised [RBS-R]; p = 0.006) and reduced anxiety (Child/Adolescent Symptom Inventory – Anxiety [CASI-Anxiety]; p = 0.048) [102].

### BIA 10-2474

Developed by Bial-Portola and the University of Porto, BIA 10–2474 (3-(1-(cyclohexyl(methyl)carbamoyl)-1H-imidazole-4-yl)pyridine 1-oxide) was a presumed reversible FAAH-selective inhibitor [103], intended to treat chronic pain, anxiety, depression, and metabolic disorders by elevating endogenous AEA [104]. In rodent studies, maximal FAAH inhibition (>90% in liver; IC_50_ of 400 nM in brain) was achieved approximately after 1 h of pre-incubation [103, 105], though later comparisons confirmed that these levels were about 2-fold less potent than PF-04457845 and 4-order of magnitude greater than JNJ-42165279 [106].

BIA 10-2474 exhibited high potency in cellular assays (in situ), blocking human FAAH activity in transfected HEK293T cells with an IC_50_ of 50 – 70 nM [107]. Preclinical toxicology studies in mice, rats, beagle dogs, and cynomolgus primates, reviewed by Hayes et al. [108], noted several incidents of animal mortality in up-titration periods evaluating the maximum tolerated dose. The animals displayed common symptoms of ataxia, weakness, decreased activity and food intake as well as weight loss. Even at doses below toxicity, signs of ataxia and weakness were still observed. Haematological effects were also reported, predominantly in mouse and rat investigations, where decreases in red blood cell count and haemoglobin were noted, along with increases in cholesterol and phospholipids, when low doses (10 mg/kg) were administered. The latter changes have been postulated to be linked to the interaction of BIA 102474 with lipid processing enzymes [107]. Evidence of axonal dystrophy, predominantly in the medulla oblongata, was also found in most species. In nearly all instances, this condition manifested as vacuolation or swelling. Furthermore, oedemas were found in various ganglia of the autonomic nervous system of rats and primates.

The majority of toxicological data was released after the first-in-human clinical trial, which resulted in deleterious health outcomes [109, 110]. The RCT aimed to evaluate the safety and tolerability of BIA 10-2474 after single and multiple oral doses, alongside the effect of food on its pharmacokinetic and pharmacodynamic properties [111]. Initial cohorts of healthy volunteers (n = 84) received either ascending single (0.25–100 mg) or multiple (2.5–20 mg for 10 days) doses, or participated in a food-interaction study (40 mg), with no adverse effects reported [109]. However, in January 2016, a cohort receiving 50 mg for 5 days developed cerebral microhaemorrhages, proving to be fatal in one participant and causing the hospitalisation of four others due to rapid progression of neurological symptoms [109]. Though the mechanism(s) of toxicity remain conjectural, van Esbroeck et al. [107] reported that BIA 10-2474 inhibition of FAAH was in fact irreversible, noting several off-target lipases potentially contributing to BIA 10-2474’s neurotoxicity through altered brain lipid metabolism. Criticism towards Bial-Portola intensified when it was revealed that key pharmacodynamic data was not disclosed before the trial [112]. This incident prompted revisions to European clinical trial regulations [113].

## CC-97489

Under clinical development by Bristol Myers Squibb (via acquisition of Celgene) is CC-97489 (also known as BMS-986368), a dual drug candidate for the treatment of epilepsy, inhibiting FAAH and monoacylglycerol lipase (MAGL), the primary enzyme responsible for the hydrolysis of 2-AG [114]. CC-97489 has been evaluated in two recently completed Phase I RCTs (i) assessing pharmacokinetics, metabolites, route of elimination, and mass balance of BMS-986368 in healthy male participants (NCT06227975, primary completion: April, 2024); and (ii) examining FAAH and MAGL availability pre- and post-treatment using positron emission tomography (NCT05065541, primary completion: January, 2024). Results from both studies have yet to be published. Bristol Myers Squibb have recently completed a third Phase I interventional RCT to evaluate the safety, tolerability, and drug levels of Multiple Ascending Doses of CC-97489 in healthy participants, including elderly and Japanese ethnicity (NCT06411730, no results available). This study presumably replaces a previously terminated Phase I safety study (NCT05099822), which was discontinued in 2022 due to a corporate strategy realignment.

### URB937

URB937 ([3-(3-carbamoylphenyl)-4-hydroxy-phenyl] N-cyclohexylcarbamate), a derivative of URB597, is a peripherally restricted FAAH inhibitor that selectively increases AEA outside the CNS [115]. Preclinical studies showed potent FAAH inhibition with notably limited CNS penetration, evidenced by ED_50_ values of 0.9 mg/kg in liver compared to 20.5 mg/kg in brain tissue [116]. URB937 exhibited analgesic efficacy in rodent models of peripheral neuropathic pain [115], alleviated acute inflammation [117] and nitroglycerin-induced migraines [118]. Furthermore, URB937 reduced bladder overactivity [119] and mitigated damage to lung tissue [120, 121]. Exxel Pharma, in partnership with the University of California, Irvine, hold the intellectual property rights focused on pain management, particularly neuropathy-related discomfort (US 9,187,413; US 9,745,255 B2 and provisional application No. 62/938,847). Phase I safety studies for oral URB937 were expected to commence in 2020; however, no clinical trials have been registered at ClinicalTrials.gov or the Australian New Zealand Clinical Trials Registry, likely reflecting delays caused by the SARS-CoV-2 pandemic.

## Conclusion and future perspectives

Cannabis, including medical cannabis, is increasingly promoted to treat diverse clinical symptoms due to ECS’s involvement in numerous physiological processes. However, this approach continues to face regulatory hurdles and demands for robust efficacy and safety, particularly concerning psychotropic effects. An alternative strategy is the development of pharmacological agents that selectively target the mechanisms of cannabis-derived compounds, offering broad therapeutic potential with higher regulatory compliance. Accordingly, the ECS has emerged as a key regulator of neurophysiological dysfunction in psychiatric disorders, with particular interest towards AEA, which modulates synaptic signalling. The development of selective, potent, efficacious and safe FAAH inhibitors represents a major pharmacological avenue to increase endogenous AEA concentrations, aimed at improving clinical symptoms and improving social and occupational functioning.

This review summarises recent developments in FAAH-selective compounds. Of the ten identified FAAH inhibitor RCTs, eight were completed, one was terminated early, and one was withdrawn due to a change in business objectives and insufficient funding. Although these inhibitors are intended for neuropsychiatric conditions such as pain, depression, anxiety and schizophrenia, most RCTs remain at Phase I, focusing on safety, tolerability, pharmacokinetics and pharmacodynamics. Only two compounds, PF-04457845 (now JZP150) and JNJ-42165279, have progressed to Phase II with mixed outcomes (Table 1). Both demonstrated pharmacodynamic target engagement, evidenced by elevated plasma and CSF AEA, yet clinical efficacy was limited. While modest efficacy was observed in cannabis dependence, neither compound showed clinical benefit for osteoarthritis pain or PTSD.

The limited clinical success of FAAH inhibitors despite promising preclinical profiles highlights the broader challenges in neuropsychiatric drug development. It raises questions about the predictive validity of preclinical behavioural models for complex, heterogeneous syndromes such as PTSD or depression. However, the lack of efficacy in PTSD may reflect its etiological heterogeneity, which arises from diverse trauma types, with resilience influenced by complex genetic and environmental factors. It is plausible that only specific subsets of individuals with PTSD may benefit from FAAH inhibitor intervention, highlighting the need for improved patient stratification. However, Mayo et al. has frequently proposed that disrupted CB_1_R signalling might underlie PTSD pathophysiology, limiting the therapeutic effects of increased central AEA concentrations [80, 95, 101].

These variable outcomes suggest that while increasing AEA holds therapeutic promise, it is unlikely to offer a universal solution for psychiatric disorders. Several trials remain ongoing or recently concluded, and their outcomes may refine our current supposition. Future RCTs should aim to improve participant characterisation for inclusion, consider variables such as type, severity and frequency/duration of trauma exposure, age, duration of illness, and cannabis consumption. Additionally, identifying predictive biomarkers, whether biomolecular or related to clinical onset, could help preselect individuals more likely to benefit from future FAAH-targeted interventions. For instance, variation at the FAAH C385A polymorphism (rs324420) may support genotype-based stratification, as C/C carriers exhibit higher FAAH activity and lower baseline AEA concentrations [38, 122], potentially conferring greater benefit from FAAH inhibition compared with C/A or A/A carriers. Complementary baseline screening of peripheral AEA and other major FAAH substrates in blood or CSF (i.e., OEA and PEA) could further identify individuals predisposed to deficient NAE tone prior to symptom onset. Neuroimaging markers may also be leveraged to screen for amygdala hyperreactivity or disrupted prefrontal limbic connectivity, common features in PTSD, depression and anxiety disorders [123–126]. Finally, clinical phenotyping could refine patient selection, noting that individuals displaying predominantly hyperarousal symptoms might yield greater benefit from AEA enhancement given its anxiolytic properties [71, 127].

A primary consideration with future FAAH-selective RCTs is balancing efficacy with safety. The fatal outcome of the BIA 10-2474 serves as a cautionary reminder of the inherent risks associated with Phase I RCTs and the absolute necessity for adhering to rigorous safety protocols and ethical standards. Furthermore, new compounds must undergo independent validation by the broader scientific community. CNS effects, though not life-threatening, were already observed in animal toxicology studies of BIA 10-2474 [108], related severe adverse events were not observed during the single ascending dose study preceding the fatal outcome. Importantly, other FAAH inhibitors assessed in RCTs to date, including PF-04457845 (now JZP150) and JNJ-42165279, have demonstrated good overall tolerability, with no reports of brain microhaemorrhages or comparable severe adverse events [82]. Adverse events in these RCTs have been typically mild to moderate. While it remains unconfirmed whether inhibition of other lipid lipases was responsible for the toxicity observed with BIA 10-2474 [107], these findings reinforce the importance of evaluating off-target effects. Non-selective FAAH inhibitors could inadvertently disrupt broader lipid networks of which the ECS is a part [13], impacting other signalling and membrane-support lipids, further emphasising the need for comprehensive safety profiles in therapeutic development. In this context, it is important to also recognise that AEA engages targets beyond CB_1_R that may contribute to its therapeutic efficacy and broader pharmacological footprint. As a CB_2_R ligand, albeit expressed at comparatively lower levels in the CNS compared to CB_1_R, AEA-CB_2_R activation may also be relevant to psychiatric disorders [128, 129]. CB_2_R signalling has been implicated in neuroimmune and neuroinflammatory processes that are increasingly recognised in psychiatric conditions [130–132]. However, the translation of CB_2_R-mediated AEA mechanisms to clinical psychiatric outcomes remains in its infancy, requiring more direct evidence. AEA also acts as an agonist at the transient receptor potential vanilloid 1 (TRPV1) ion channel [133, 134], which has been proposed as a potential therapeutic target in anxiety, depression, and psychosis [135]. As aforementioned, FAAH is also the principal enzyme responsible for OEA and PEA hydrolysis, with its inhibition linked to elevation of these NAEs. This potentially expands the lipid mediator network of targets, including PPAR-α, PPAR-γ, GPR55, and GPR119, through which OEA and PEA act as ligands [134, 136–138].

Despite extensive preclinical characterisation, clinical investigations into URB597 remain limited. This may partly reflect delays due to licensing issues following multiple corporate acquisitions [139] and safety concerns after the BIA 10-2474 incident, which may have intermittently detracted pharmaceutical interest towards FAAH irreversible inhibitors. Irreversible inhibition can extend enzyme inhibition beyond the elimination half-life, increasing the risk of idiosyncratic drug-related toxicity [140]. In the BIA 10-2474 incident, FAAH inhibition was maintained for over 72 h at doses above 2.5 mg, although drug exposure was designed to last between 4–6 h [141]. In contrast, URB597 has a relatively short half-life (~2 h) [70], and its ability to avoid competition for FAAH activity with endogenous ligands (i.e., AEA, OEA, PEA) may enhance potency, favouring lower doses and reducing the risk of ceiling concentration effects [142].

Future investigations into the second FAAH isoform (FAAH-2) may offer additional therapeutic avenues. Although in vitro assays demonstrated FAAH-2 accounts for less than 10% of the relative rate of AEA hydrolysis, compared to FAAH [62], intact cell studies with [^14^C]-AEA indicated that FAAH-2 can achieve hydrolysis rates up to 40% of FAAH’s efficiency [67]. Moreover, FAAH-2’s distinct membrane topology, oriented toward the luminal compartment of the endoplasmic reticulum and localising to lipid droplets, raises the possibility of isoform-specific inhibitors targeting distinct AEA pools [62, 67, 143, 144]. However, its limited expression in the brain and predominance in peripheral tissues [62, 143, 145, 146] may restrict its relevance for neurobiological applications. Additionally, the absence of FAAH-2 in lower order mammals complicates preclinical research using conventional animal models [147].

Although beyond the scope of this review, the therapeutic potential of monoacylglycerol lipase (MAGL) inhibitors, responsible for the hydrolysis of 2-AG (Fig. 1), warrants mention. Preclinical studies using JZL-184 [82] have shown that elevated cerebral concentrations of 2-AG are associated with anxiolytic, antinociceptive, anti-inflammatory properties, and anti-depressive-like effects [148–150]. However, a Phase II RCT for the MAGL inhibitor Lu-Ag06466 (formerly ABX-1431) in Tourette Syndrome and Chronic Motor Tic Disorder (NCT03625453) failed to meet its primary endpoint, though benefits were noted in participants with comorbid attention deficit hyperactivity disorder (ADHD) [151]. Additionally, CNS-related, treatment-emergent side effects have been reported in both preclinical and clinical settings [151, 152]. Dual FAAH/MAGL inhibitors are also under investigation. Preclinical studies into inhibitor JZL-195 [153] have demonstrated efficacy in models of traumatic brain injury, migraine-like pain and sporadic Alzheimer’s disease [154–156]. However, such dual inhibition represents a potent ECS intervention that may limit therapeutic applicability. Currently, no clinical outcomes are available for CC-97489, the only dual FAAH/MAGL undergoing RCTs.

AEA’s agonist activity toward TRPV1 (“endovanilloid” signalling) has also been considered, based on the premise that combining FAAH inhibition with TRPV1 blockade might shift elevated AEA activity away from TRPV1-mediated effects toward CB_1_R-directed signalling [157]. Observed in animal models, the dual approach has been associated with antidepressant-like effects (forced swim test) [158–160], reduced anxiety-like behaviour (elevated plus maze) [161], and inhibition of contextual fear memory [162]. However, to our knowledge, no RCTs have been conducted, to date, that employ dual FAAH/TRPV1 inhibitors.

In summary, FAAH inhibitors have not yet fulfilled their clinical promise that arose from preclinical data. Ongoing and future clinical trials are critical in overcoming the current challenges and clarifying their role in psychiatric treatment. Careful patient stratification, precision psychiatry approaches, rigorous safety and selectivity evaluation will be essential to unlock the full therapeutic potential of FAAH-targeted interventions in mood, anxiety, and trauma-related disorders.

## Data Availability

Data availability is not applicable to this manuscript as no new data were created or analysed in this study.
